# Distributional effects of technological regime changes: hysteresis, concentration and inequality dynamics

**DOI:** 10.1007/s43253-021-00052-5

**Published:** 2021-10-22

**Authors:** Herbert Dawid, Jasper Hepp

**Affiliations:** 1 Department of Business Administration and Economics and Center for Mathematical Economics, Bielefeld University, Universitätsstraße 25, 33615 Bielefeld, Germany; 2 Bielefeld Graduate School of Economics and Management (BiGSEM), Bielefeld University, Universitätsstraße, 33615 Bielefeld, Germany

**Keywords:** Agent-based model, Technological regime, Inequality, Firm polarization, C63, E24, J24

## Abstract

In this paper, we study the effect of different types of technological regime changes on the evolution of industry concentration and wage inequality. Using a calibrated agent-based macroeconomic framework, the Eurace@Unibi model, we consider scenarios where the new regime is characterized by a finite time period of more frequent respectively more substantial changes in the frontier technology compared to the old regime. We show that under both scenarios, the regime change leads to an increase in the heterogeneity of productivity in the firm population and to increased market concentration, where effects are much less pronounced if the new regime differs from the old one with respect to the frequency of innovations. If the new regime is characterized by an increase of the size of the frontier jumps along the technological trajectory, the evolution of the wage inequality has an inverted U-shape with a large fraction of workers profiting in the very long run from high wages offered by dominant high-tech firms. Finally, it is shown that (observable) heterogeneity of worker skills plays an important role in generating these dynamic effects of technological regime changes.

## Introduction

Increasing polarization in the last decades is a major trend in OECD economies. On the worker-side, wages between high and low educated employees diverge (Autor et al. 2003; Autor et al. 2020b), employment dynamics polarize in a U-shaped pattern across skill groups (Goos and Manning 2007; Goos et al. 2014; Autor and Dorn 2013) and income for top earners further pulls away (Atkinson et al. 2011; Saez and Zucman 2020). On the firm-side, adoption rates of new technologies are uneven and firms disperse further in terms of productivity (Andrews et al. 2016; Berlingieri et al. 2017) and skills (Card et al. 2013; Song et al. 2019), market shares are increasingly allocated to a few large firms (Autor et al. 2020a) and returns to capital are more and more unevenly distributed (Furman and Orszag 2015).^1^ At the same time, recent evidence points to an interconnection of the phenomena at the worker- and firm-level. In particular, the availability of matched employee-employer datasets has brought about a new strand of empirical results focusing on the role of the firm in shaping inequality dynamics and labour market outcomes. Starting from the work by Abowd et al. (1999), the evidence overwhelmingly points to an important role played by firm heterogeneity (Bagger et al. 2010; Faggio et al. 2010; Card et al. 2013; Barth et al. 2016; Song et al. 2019; Criscuolo et al. 2020; Bormans and Theodorakopoulos 2020). For example, Song et al. (2019) assess that two-third of the rise in wage inequality from 1978 to 2013 in the USA can be explained by between-firm differences.

A natural question arising in this respect regards the main driver of this pervasive polarization. Several studies focus on firms’ investment in IT capital and link productivity dispersion (Dunne et al. 2004; Faggio et al. 2010) or market concentration (Bessen 2020) to heterogeneous ICT adoption rates. Inline with this, Andrews et al. (2015), Andrews et al. (2016), and Akcigit and Ates (2019) find a slowdown in technology diffusion from leader to laggard firms. Others link the widening gap between firms to the rise of intangible capital (Crouzet and Eberly 2018). As a consequence, these different investment rates into software or IT across firms have then contributed to the dispersion in wages (Faggio et al. 2010; Barth et al. 2020).

The agenda of this paper is to improve our understanding of two so far less studied aspects of the relationship between technological change, industry dynamics and evolution of income inequality. In particular, we focus on the differences between short- and long-run effects of a technological regime change and examine how these effects depend on the type of technological evolution emerging under the new regime. More precisely, we distinguish between two technological regimes. First, a regime under which the frontier moves in many relatively small steps, such that producers using the technology when investing can select from a large choice of close to the frontier instances of the technology, which we refer to as the *Frequency* scenario. Second, we consider a regime characterized by less frequent and larger jumps in the frontier technology, labelled as *Increment* scenario. Referring, e.g. to Malerba and Orsenigo (1997), one can think of the software industry as an example of a *Frequency* scenario, since ‘*... opportunity conditions are very high with a wide variety of potential technological approaches and solutions ... Therefore, one would expect specialization ... with many innovators.*’[p.113]. On the contrary, Malerba and Orsenigo (1997) describe the computer industry characterized by ‘*... high technology opportunities with limited technological variety … Therefore, one would expect few innovators to be present in the industry ...*’ [p.113] and in particular, if we consider the transformative effects of technological breakthroughs like touch-screens for tablet computers, this industry can be considered as an example of what we refer to as the *Increment* scenario. Also, the distinction between exploration and exploitation by March (1991) gives rise to different technological dynamics represented by our two scenarios. Here, the *Increment* scenario corresponds to technological change mainly driven by exploration, i.e. a process where firms try to find fundamentally new approaches for designing or producing a certain product. Since many of the explored new approaches do not result in solutions dominating the current frontier, such a regime leads to rare productivity jumps at the frontier, which however tend to be large. Innovation processes mainly focusing on exploitation of existing technologies, on the other hand, lead to smaller but more frequent improvements in the technological space and hence correspond to our *Frequency* scenario.

For both scenarios in our setting, the change in the technological regime leads to a temporary acceleration of productivity growth, which then eventually flattens out again. Such a process might be induced by a *technological paradigm shift* as it has been described by Dosi (1982) and Perez (2010). As has been argued in this literature, it should be expected that the technological trajectory emerging after such a shift flattens after some time such that the growth rate returns to a value similar to that before the arrival of the new regime. Nevertheless, innovations such as the computer or today’s digital technologies affect the development of the some industries in a long-lasting way (Freeman 2009; Knell 2021). In our setting, the speed of technological change is increased for a finite time interval after the shift; either by increasing the frequency of new innovations arriving or by increasing the productivity jump a single innovation gains. As we will see in Section 4, in setting the economic conditions under which firms make their investment decisions, the technological environment influences firm’s behaviour in line with the evolutionary tradition (Malerba and Orsenigo 1993).

In our distinction between these scenarios, we use a reduced form representation of the change of the technological basis of the production process in the sense that we assume that the technological developments are fully embodied in the capital used for production and increase the productivity of the production process. The upstream sectors offer their downstream buyers different palettes of capital goods which evolve over time due to technological change. The main research question we address in this setting is whether the type of change of the technological frontier—Increment vs. Frequency—has systematic implications for the medium- and long-run dynamics of industry concentration and inequality even if the average productivity growth rate is the same in both scenarios. Furthermore, we want to improve our understanding of crucial mechanisms responsible for the dynamics emerging in these two scenarios.

We carry out our analysis within the agent-based Eurace@Unibi model (Deissenberg et al. 2008; Dawid et al. 2019), which captures the interplay of the labour, capital and goods market as well as the endogenous diffusion of new technologies in the firm population and the updating of worker (specific) skills due to on-the-job learning. Furthermore, the model captures complementarity on a firm level between the specific skills of the employees and the quality of the installed capital goods, as well as the heterogeneity of workers with respect to general skills (e.g. level of education), which determine the speed by which workers are able to improve their specific skills. These properties make the model particularly suitable for addressing our research questions. Our experimental setup to investigate the channel from technological change to inequality relies on a variation in the technological frontier (the most productive capital good offered to firms). In our setting, the dynamics of the frontier is determined by the number of innovations in a given time period and the factor by which the productivity of a new vintage improves on the previous frontier technology. Starting from a baseline scenario with a fixed trajectory over the whole simulation run, we model the *Frequency* scenario by assuming that productivity increasing innovations arrive more frequently than in the baseline, whereas in the *Increment* scenario, the frequency of innovation remains unchanged but the average productivity increase of each innovation is larger. Both scenarios share the same average productivity growth rate. This variation in the trajectory of the frontier occurs for a given amount of time, after which in all scenarios technological growth returns to the baseline values.

The main insights from our analysis are that temporary technological regime change has both short and long run effects on concentration and inequality and that these effects indeed differ qualitatively between the the *Frequency* and the *Increment* scenario. In the short run, the acceleration of technological change leads to a somehow larger productivity dispersion across firms and associated with this an increase in wage inequality. Allocation of worker skills across firms and market concentration is however hardly affected. These effects are qualitatively the same under the *Frequency* and the *Increment* scenarios. The key differences between the effects of technological regime change under the *Frequency* and the *Increment* scenarios emerge only in the long run, where under the *Increment* scenario a bimodal firm distribution with respect to productivity and (specific) skills of workers emerges. Technological laggards face growing unit labour costs, which reduces their competitiveness and make their market shares shrink. This reinforces market concentration up to a point where all general skill groups in the population profit from the high productivity of the high tech firms. Hence, the economy approaches a state with very high concentration, but decreasing wage inequality. Under the *Frequency* scenario, such a self-reinforcing concentration dynamic does not arise. If workers all have the same general skill level, only a strongly reduced concentration effect appears under any of the scenarios, which shows that the mechanism crucially relies on the fact that the speed of specific skill acquisition is heterogeneous between workers and that high productivity firms can explicitly target fast learning workers (those with high general skills), which on average also have higher specific skills. Finally, we find patterns of hysteresis, i.e. long run effects of the temporary change in the frontier dynamics, since even after the return to baseline values, the long-run market concentration and inequality dynamics differs depending on which technological regime was active during the period of accelerated technological change. We show that also this path dependency on the aggregate level is driven strongly by the heterogeneous skill distribution.

Our setup is related to models that incorporate a technological revolution in settings with labour that is heterogeneous in the ability (or costs) to employ the new machines, such as in Greenwood and Yorukoglu (1997) or Caselli (1999). Accelerated technological change leads to uneven adoption rates of technologies under skill differences among firms and a shift in demand to high ability workers, which in turn increases wage inequality. On the other hand, the mechanism in our model leading to further skill segregation is found in sorting models such as Kremer and Maskin (1996). However, in contrast to our model, the segregation in Kremer and Maskin (1996) is initiated by negative productivity spillovers from low- to high-skilled workers. The higher the complementarity between the different tasks performed by the heterogeneous workers, the stronger the divergence among firms. In our model, segregation is not driven by the organizational setup within the firm, but rather by the feedback loops of firms decisions on labour and capital market reinforcing and amplifying the dispersion. Overall, these mechanisms are inline with aforementioned empirical findings (Dunne et al. 2004; Faggio et al. 2010), which document a link between increasing productivity and wage dispersion on the firm-level.

Related to this paper is the canonical task–based model as in Acemoglu and Autor (2011), which offers an explanation on labour market polarization (Autor et al. 2003; Goos et al. 2014). The model in this paper is different in two ways. First, all skill groups are complementary towards capital goods in the Leontief production function and hence, we do not allow for substitution between capital and labour and rely on a fixed capital to labour ratio. Second, we focus on the role of the endogenously evolving industry structure in shaping the labour market under different technological regimes. So far, the theoretical literature has devoted little attention to the impact of firms and competition in shaping polarization on the labour market. The canonical model as in Acemoglu and Autor (2011) is based on a representative firm and only distinguishes between different tasks. In contrast, within the Eurace@Unibi model firms compete and are heterogeneous in productivity. Irrespective of their general skill all workers can be employed and matched with any machine where the advantage of a higher skill level lies in the ability to learn faster during the endogenous formation of the specific skills on the job.

The paper is part of a growing literature applying agent-based models to macroeconomic analysis (Dawid and Delli Gatti 2018), labour markets (Neugart and Richiardi 2018) and innovation economics (Dawid 2006). Closely related is Hepp (2021), in which a similar setup is used to investigate the effect of an acceleration in technological change on firm-level determinants of the largest emerging firms. Other previous publications relying on the Eurace@Unibi model focus not only on policy analysis in different areas such as regional cohesion (Dawid et al. 2014; 2018b), banking regulations (van der Hoog and Dawid 2019), fiscal stabilization (Harting 2021), de-unionisation (Dawid et al. 2021), optimal containment policies during the COVID-19 crisis (Basurto et al. 2020), but also on the diffusion of competing technologies in the context of climate change (Hötte 2021) or the role of social networks for inequality dynamics (Dawid and Gemkow 2014). Issues related to inequality dynamics and labour market polarization as well as its interplay with technological change have been studied also in the framework of several other agent-based macroeconomic frameworks. Most prominently, the K+S model has been developed to investigate the interconnection of technological and inequality dynamics (Dosi et al. 2017; 2020a, b). In a recent publication (Dosi et al. 2021), the model is extended to distinguish between endogenously arriving incremental and radical innovations in the upstream sector, whereas the later creates new downstream sectors with a higher product complexity. The focus of the analysis is on the dynamics of labour demand and its interplay with consumption patterns. An earlier extension of the K+S model by Mellacher and Scheuer (2020) incorporates heterogeneous worker types and investigates the skill biased technological change hypothesis in a setting with endogenous technological change. Silva et al. (2012) find increasing wage inequality in a setting with unbiased technological change and labour market frictions stemming from an endogenous matching process. Caiani et al. (2019) show that higher wages for lower skilled workers leads to stronger economic growth in an agent-based model with a segmented labour market, heterogeneous propensities to consume and endogenous innovation rates. Terranova and Turco (2021) present an agent-based model to investigate the dynamics of concentration and stagnation emphasising the importance of technological knowledge accumulation as a source of market power. Finally, Bertani et al. (2020a, b) introduce intangible capital in the Eurace model and investigate the dynamics of increasing returns as well as technological unemployment due to increased investment into the new technologies. The specific contribution of our paper relative to this literature is that we focus on short and long run implications of a temporary acceleration of the speed of the technological frontier and also that we explicitly analyse how the type of technological change, driven by few large innovations or by many small ones, influences the industry dynamics and the evolution of wage distribution. In particular, the consideration of the joint dynamics of endogenous skill adjustment by workers and firm decisions about adoption of newest technology vintages allows us to obtain new insights into the process of innovation driven growth in such a setting.

The paper is organized as follows. In Section 2, we sketch the model and in Section 3, we describe the experimental setup for this paper. Results and discussion are given in Section 4 and we conclude in Section 5. Technical details such as the parameter choices as well as results of statistical tests can be found in the Appendix A–B.

## The model

### An overview

The Eurace@Unibi model is a closed macroeconomic agent-based model consisting of one capital good producer, populations of consumption good firms and households and a labour, consumption good and capital good market. In addition, the model contains banks, one central bank and a credit market.^2^ A particular focus in this paper lies on the diffusion of technology and its effect on economic dynamics. Vintages heterogeneous in quality and prices are offered by the capital good producer. New vintages arrive with an exogenous arrival rate and improve the offered productivity by a fixed increment. Consumption good firms purchase these investment goods and combine them with heterogeneously skilled labour to produce products that households purchase. Workers are equipped with a general skill level, which is observable on the labour market and signals the worker’s ability to improve her (ex ante unobservable) specific skill. Complementarity between workers’ specific skills and the quality of physical capital implies that the actual productivity of a worker in a firm is determined by the minimum between the specific skill of the worker and the (average) quality of the machines used by the firm. Due to on-the-job learning, a worker increases her specific skill level. The learning is faster the higher the worker’s general skill and the larger the gap between the productivity of the machine and the specific skill level of the worker. When investing, firms choose which capital vintage to acquire based on a heuristic taking into account the expected future productivity of the vintage in the firm, which positively depends on the average general skill level of the firms’ employees, and the price of the vintage. The basic time unit in the Eurace@Unibi model is interpreted as one day, where each month consists of 20 (working) days and therefore a year has 240 time units.

The way technological change is captured in the model allows us to investigate the endogenous diffusion of technologies under the *Increment* and the *Frequency* scenario characterized by different frequencies of the arrival of new vintages and the size of the quality increase associated with such innovations. The built-in feedback loops between technology-side and worker-side are of particular relevance during the process of technology diffusion, and the model setup enables us to gain a rich understanding of the underlying mechanisms for each technological regime.

Since this paper uses the benchmark version of the Eurace@Unibi model as fully described in Dawid et al. (2019), in the following subsection we only sketch the parts of the model which are most crucial for the results presented here and refer the reader for more details to previous publications, in particular Dawid et al.(2014; 2018a; 2019).

### Agents and markets

#### Capital good firm

One monopolistic capital good producer offers at each point in time a set of vintages {1,..,*V*_*t*_} with different productivities *A*^*v*^ and prices *p*_*v*_ for all *v* ∈{1,..,*V*_*t*_} with infinite supply. The technological frontier $A^{V_{t}}$ – representing the productivity of the most productive vintage *V*_*t*_ – develops over time. More precisely, we stochastically determine a sequence ${\mathscr{T}} = \{\tau _{i}\}, i=1,2,..$ with *τ*_*i*_ < *τ*_*i*+ 1_ of innovation times in [−*T*,*T*], where 2*T* denotes the total duration of our simulation run and we ignore the first *T* periods as a burn-in phase.^3^ In the baseline setting, a new vintage is introduced at every point in time *τ*_*i*_ and this new vintage is added to the set of offers, i.e. $V_{\tau _{i}+1} = V_{\tau _{i}} + 1$, where the productivity of the new vintage increases by a factor (1 + *Δq*_*inv*_) > 1 compared to the previous frontier vintage. Hence, the productivity of the frontier can be written as

1 $$ \begin{array}{@{}rcl@{}} A^{V_{t}} = (1+{{{{\varDelta}}}} q_{inv} )^{i} \cdot A^{V_{-T}} \quad \text{if} \tau_{i} < t \leq \tau_{i+1} \end{array} $$

with $A^{V_{-T}}$ the productivity of the best vintage at the beginning of the burn-in phase. The trajectory of the frontier is determined by two parameters: (1) the number of innovations in a given time interval and (2) the increment *Δq*_*inv*_ increasing the productivity of the current frontier from a single new innovation. This rather simplistic modelling of the capital good sector as the locus of technological change allows us to investigate variations in the number of innovations in a given time interval and in the size of the productivity jump *Δq*_*inv*_, distinguishing between the *Increment* and *Frequency* scenarios (see Section 3 for more details).

#### Consumption good firms: production

Firms produce horizontally differentiated consumption goods in a Leontief type production function with labour and capital as inputs. The capital stock *K*_*i*,*t*_ consists of different vintages *v* with different productivities *A*^*v*^. Each stock follows

2 $$ \begin{array}{@{}rcl@{}} K_{i,t+1}^{v} = (1-\delta) \cdot K_{i,t}^{v} + I_{i,t}^{v} \end{array} $$

with investment $I_{i,t}^{v}$ and depreciation rate *δ*.

Output *Q*_*i*,*t*_ is produced by combining labour *L*_*i*,*t*_ with capital *K*_*i*,*t*_ in a Leontief production function. Labour and capital are also complementary in the determination of the productivity of the firm, given by *min*[*A*^*v*^,*B*_*i*,*t*_]. This yields the production function

3 $$ \begin{array}{@{}rcl@{}} Q_{i,t} = \sum\limits_{v=1}^{V_{t}} min \ \left[ K_{i,t}^{v}, \ max \left[0, L_{i,t} - \sum\limits_{k=v+1}^{V_{t}} K_{i,t}^{k}\right] \right] \cdot min\left[A^{v}, B_{i,t}\right] \end{array} $$

with *A*^*v*^ the productivity of vintage *v* and *B*_*i*,*t*_ the average specific skills within the firms’ workforce.

To plan the output level, an estimated demand function is calculated once a year based on past data. Production takes place once a month. In case of expansion, firms get active on the capital as well as labour market. Afterwards, firms deliver their products to the consumption goods market, where they are stored and purchased by households. Firms aim to keep a stock of goods to satisfy demand over the whole month and thus produce above the expected sales by adding a buffer.

#### Consumption good firms: pricing

Closely related to the production planning is the price setting, which is based on the *management science* approach as described in Dawid and Harting (2012). Firms set prices once a year based on *simulated* *purchase* *surveys* with households. Comparing across products, a subset of households sends their willingness to purchase the product of the firm conditional on a given price. Firms choose the profit maximizing option among the considered prices given the resulting demand calculations and their production planning as well as cost structure.

#### Households

Workers *h* hired by firms differ with respect to their human capital endowment. Each has a fixed and exogenous general skill $b^{gen}_{h} \in \{1,2,3\}$ reflecting her educational level, with $b^{gen}_{h} = 1$ the lowest and $b^{gen}_{h} = 3$ the highest. In addition, workers are equipped with an endogenously evolving specific skill *b*_*h*,*t*_ reflecting experience on the job. General skills are observable during the hiring process on the labour market, while specific skills are only revealed to firms during production. When worker *h* is employed by a firm with average quality of the capital stock *A*_*i*,*t*_, the specific skill level of the worker is adjusted according to:

4 $$ \begin{array}{@{}rcl@{}} b_{h,t+1} = b_{h,t} + \chi\left( b^{gen}_{h}\right) \cdot max\left[0, A_{i,t}-b_{h,t}\right] \end{array} $$

with $0 < \chi \left (b^{gen}_{h}\right ) < 1$ denoting the speed of learning for the worker’s general skill group $b^{gen}_{h}$.^4^ The value $\chi \left (b^{gen}_{h}\right )$ is increasing in general skills, reflecting that learning is faster the higher the educational level of the worker (see Table 3 in Appendix Appendix). It should be noted that workers with different general skills differ only with respect to their learning rate, but otherwise can be fully substituted for each other.

#### Consumption good firms: Vintage choice

Investment into new vintages happens only when firms are not able to produce their desired output with their current capital stock. Capital demand is estimated by taking the gap in output the firm cannot produce at the moment and is adjusted with firms’ average productivity.

To choose between vintages *v* offered by the capital good producer, firms calculate an effective productivity $\hat {A}^{eff}_{i,t}(v)$ taking into account their average specific skills *B*_*i*,*t*_ within their workforce over a fixed time horizon *S*:

5 $$ \begin{array}{@{}rcl@{}} \hat{A}^{eff}_{i,t}(v) = \sum\limits_{s=t}^{S} \left( \frac{1}{1+\rho} \right)^{s} \cdot \min \left[A^{v}, \hat{B}_{i,t+s}(A^{v}) \right] \end{array} $$

with *ρ* the discount rate. To obtain an estimation for the expected specific skill $\hat {B}_{i,t+s}$ in period *t* + *s* firms take into account the current average general skills $B_{i,t}^{gen}$ within their workforce:

6 $$ \begin{array}{@{}rcl@{}} \hat{B}_{i,t+s} = \hat{B}_{i,t+s-1} + \chi \left( B_{i,t}^{gen}\right) \cdot \max \left[ A^{v} - \hat{B}_{i,t+s-1}, 0 \right]. \end{array} $$

Taking this into account, firms choose from the set of currently available vintages *V*_*t*_ according to a logit-choice model, in which the effective productivity as well as the price of each vintage is considered. A vintage *v* ∈{1,..*V*_*t*_} is selected with the probability

7 $$ \begin{array}{@{}rcl@{}} \mathbb{P}[\text{Firm i selects vintage \textit{v}}] = \frac{exp \left( \gamma^{v} \ \ln \left( \frac{\hat{A}^{eff}_{i,t}(v)}{{p_{t}^{v}}} \right) \right) }{{\sum}_{\bar{v}=1}^{V_{t}} \ exp \left( \gamma^{v} \ \ln \left( \frac{\hat{A}^{eff}_{i,t}(\bar{v})}{p_{t}^{\bar{v}}} \right) \right)}. \end{array} $$

This implies that firms do not necessarily pick the frontier technology. If the effective productivity of the best-practice vintage does not offset its higher price, the firm rather invests in a less productive but cheaper capital good. Hence, the current average level of general skills in the firm’s workforce, which influences the effective productivity of the different vintages has an important influence on the vintage choice of the firm.

#### Labour market

The labour market consists of two rounds of a search-and-matching procedure. In short, consumption good firms post vacancies on the labour market to which households apply excluding the offers below their own reservation wage.

In the process of production planning firms estimate their labour demand accordingly. Starting with lower skilled workers, firms fire if their workforce is too large and the desired output can be produced with less labour. In case a firm needs to hire more workers, a wage offer is posted on the labour market. The wage offer $w_{i,t,g}^{o}$ sent out to each skill group *g* is composed of two parts:

8 $$ \begin{array}{@{}rcl@{}} w_{i,t,g}^{o} = w^{base}_{i,t} \cdot min\left[A_{i,t}, \bar{B}_{i,t-1,g}\right]. \end{array} $$

The rationale underlying this wage offer of the firm is that it multiplies the wage it is willing to pay per productivity unit, which we label as base wage offer ($w^{base}_{i,t}$), with an estimate of the productivity of the applicant. The base wage offer $w^{base}_{i,t}$ is driven by the market tightness and is adjusted upwards by a factor (1 + *φ*) if a firm has more than $\bar {v}$ unfilled vacancies at the end of the hiring cycle. The second expression in Eq. 8 gives the expected productivity of a worker *h* with general skill *g* in the firm. Since firms do not observe the specific skill of an applicant, they estimate that skill using the average specific skills $\bar {B}_{i,t-1,g}$ of their current employees with the same general skill level *g* as the applicant. In light of the complementarity between worker specific skills and the quality of the firm’s physical capital, the expected productivity of an applicant with such specific skill is given by $min\left [A_{i,t}, \bar {B}_{i,t-1,g}\right ]$.

Unemployed workers consider a random subset of wage offers for their skill group restricted by their reservation wage $w_{h,t}^{R}$ as a lower bound. The level of the reservation wage is determined by their previous wage when entering unemployment, and afterwards is adjusted downwards by a factor *ψ* < 1 in each period of unemployment. The lower bound is given by the unemployment benefit payment calculated as *u* percentage of their previous wage. In a next step, unemployed workers send their applications to a set of chosen offers and firms decide on the application ranking workers with high general skills above low-skilled applicants. Finally, workers accept the highest offer. This whole cycle is passed through twice before the labour market closes.

#### Consumption good market

Consumption good firms offer their product at posted prices. Households use a buffer stock rule to determine their consumption budget under consideration of their (current) income and their savings. They choose the consumption good firm from which to buy using a logit-choice model, where the probability to buy from producer *i* is given by

9 $$ \begin{array}{@{}rcl@{}} \mathbb{P}[\text{Household h selects product \textit{i}}] = \frac{exp(- \gamma^{c} \ \ln(p_{i},t))}{{\sum}_{i^{\prime}} exp(- \gamma^{c} \ \ln(p_{i}^{\prime},t))} \end{array} $$

with parameter *γ*^*c*^ denoting the price sensitivity of consumers. This formulation captures in reduced form that the consumers’ product choice might be influenced not only by the price but also by individual preferences and other factors not explicitly captured in the model.

#### Firm bankruptcy, exit and entry

Firms have access to credit from banks, but depending on the financial standing of the firm and the bank might be rationed on the credit market. In this case, a firm might have to declare bankruptcy and to go out of business. It stops all productive activities and all employees lose their jobs. The firm writes off a fraction of its debt with all banks with which it has a loan and stays idle for a certain period before it becomes active again. Apart from these mechanisms, there is no exit or entry of firms in the model.

#### Government

The government collects income and profit taxes in order to finance the unemployment benefits. Tax rates are adjusted over time such as to target a balanced budget.

### Parametrization

For our simulation experiment we use a standard constellation of parameters (see Tables 2 and 3 in Appendix Appendix), which has been determined in a combination of a direct estimation process and an indirect calibration and has been used in several previous studies based on the Eurace@Unibi model (see Dawid et al. (2018a; 2019)). As demonstrated, e.g. in Dawid et al. (2018b; 2019) the model is able to reproduce a wide range of empirical stylized facts on an aggregate level, like growth patterns and business cycle properties, as well as on more disaggregated levels, such as properties of firm distributions or labour market regularities.

## Experimental setup

The main goal of our analysis is to shed light on the interplay of technological change and inequality as well as on the underlying firm-level dynamics and mechanisms. In our simulation experiment, we vary the type of technological change by alternating the trajectory of the technological frontier.

We implement the shift in the technological regime by assuming that the average growth rate of the frontier technology in a given time interval [0,*T*^*reg*^] with *T*^*reg*^ < *T* is substantially higher compared to that in the baseline. More precisely, we distinguish between two scenarios differing with respect to the driver of accelerated technological change in the new regime. First, in the *Frequency* scenario, we increase the number of innovations in the interval [0,*T*^*reg*^] by a factor 3 compared to the baseline. Denoting by *n*^*B*^ the number of innovation times *τ*_*i*_ ∈ [0,*T*^*reg*^] in ${\mathscr{T}}$, we generate a new sequence of innovation time ${\mathscr{T}}^{F}$ by adding 2*n*^*B*^ stochastic innovation times in [0,*T*^*reg*^] to the sequence ${\mathscr{T}}$. The trajectory under the *Frequency* scenario follows the sequence ${\mathscr{T}}^{F}$ of innovation times keeping the increment at the baseline value (${{{{\varDelta }}}} q_{inv}^{F} = {{{{\varDelta }}}} q_{inv}$). The second scenario, called *Increment*, increases the multiplier of the productivity increment to a value ${{{{\varDelta }}}} q_{inv}^{I} > {{{{\varDelta }}}} q_{inv}$ keeping the set of innovations times at the baseline sequence ${\mathscr{T}}$. To be able to compare the two scenarios properly, we choose ${{{{\varDelta }}}} q_{inv}^{I}$, such that the productivities of the frontiers at *T*^*reg*^ are identical under the two types of new technological regimes. We denote the time where the regime shift occurs, as year 0 and all scenarios share the same set of innovation times in the burn-in phase before that point in time. In line with the literature on technological trajectories (Dosi and Nelson 2010), we assume that the additional growth potential of the new regime disappears over time. We consider simulation runs over 40 years (*T* = 9600), excluding the burn-in phase, and assume that the accelerated technological change last for 20 years (*T*^*reg*^ = 4800). In all scenarios, the innovation times for *t* > *T*^*reg*^ are again given by the same sequence ${\mathscr{T}}$ such that the technological growth rate returns to the value of the *Baseline* scenario. In Table 1, we summarize the parameters describing the technological frontier for each scenario.

**Table 1:** Setup for the technological frontier between years 0 to 20 (*T*^*reg*^ = 4800)

	Baseline	*Frequency* scenario	*Increment* scenario
Number of innovations in [0,*T*^*reg*^]	8	24	8
Productivity increase *Δq*_*inv*_	0.025	0.025	0.076890625

**Fig. 1:**
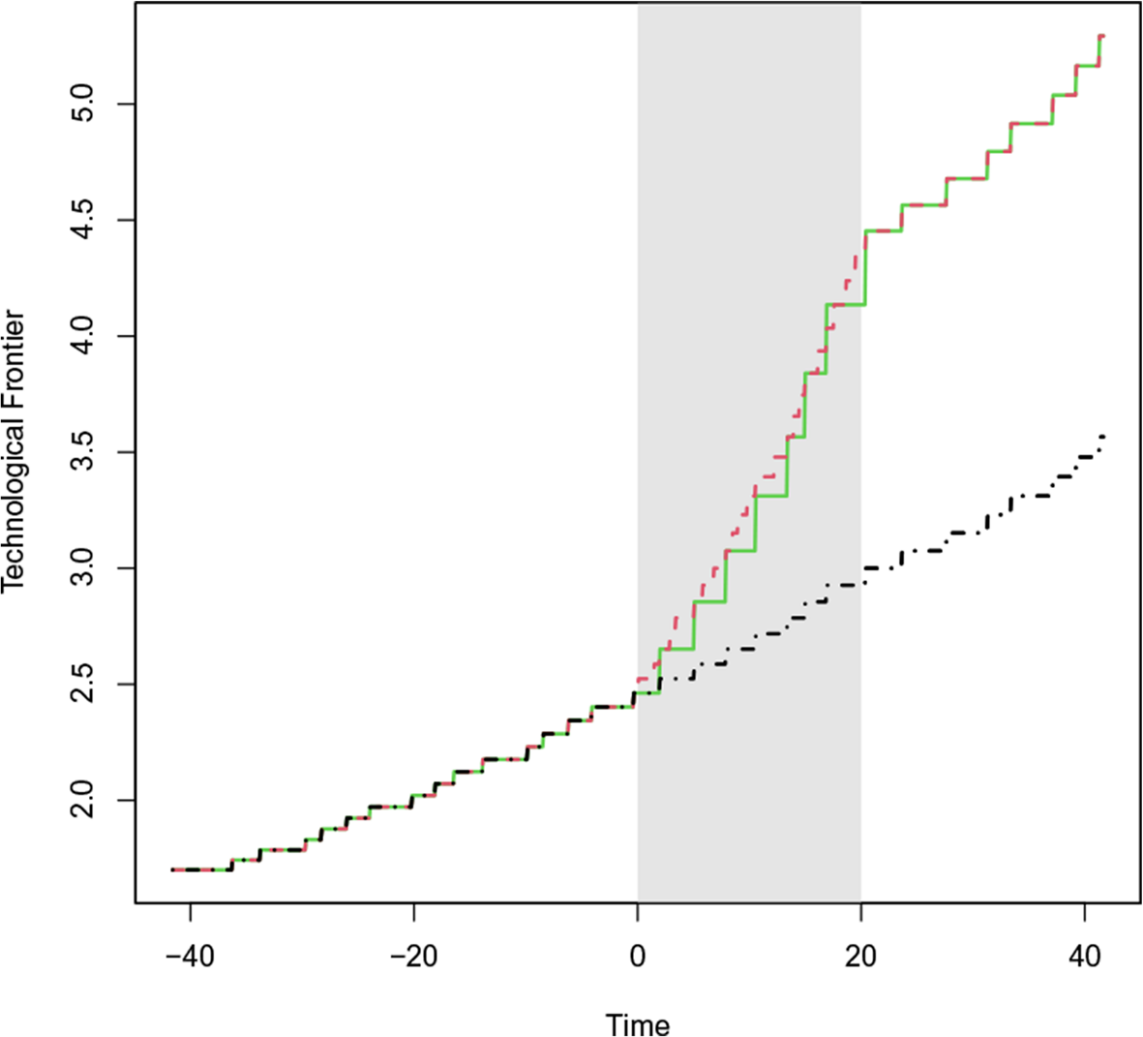
Technological frontier $A^{V_{t}}$ (the most productive vintage in time *t*). Grey area indicates the regime shift. *Baseline* in black, *Frequency* in red and *Increment* in green

Figure 1 shows the technological frontier for all three scenarios. The black line gives the most productive vintage at each point in time for the *Baseline* scenario. In red, we show the *Frequency* and in green, the *Increment* scenario. The grey area indicates the time of acceleration in technological change. Afterwards, both scenarios return to the initial values and grow in parallel to the *Baseline* scenario. The figure clearly shows that both scenarios arrive at the same point in year 20.

In the following section, we derive our results from a comparison across all three scenarios with particular focus on the difference between the *Frequency* and the *Increment* one. We will highlight our key findings by stating them as *Observations*. In the analysis, we focus on the effect of the shift in the technological regime and abstain from showing the dynamics in the time span before the regime change. However, we are interested to study not only the effects of the regime change during years 0-20, when the frontier growth is accelerated, but also in the long run, i.e. in the years after the growth rate of the trajectory has returned to its baseline level. Hence, all following figures show the years 0 to 40. In order to capture the stochastic nature of the dynamics emerging in our model, we carry out batches of 40 simulation runs for each considered scenario. Table 4 in Appendix Appendix documents the results of statistical tests confirming the statistical significance of the our observations.

## Economic analysis

**Fig. 2:**
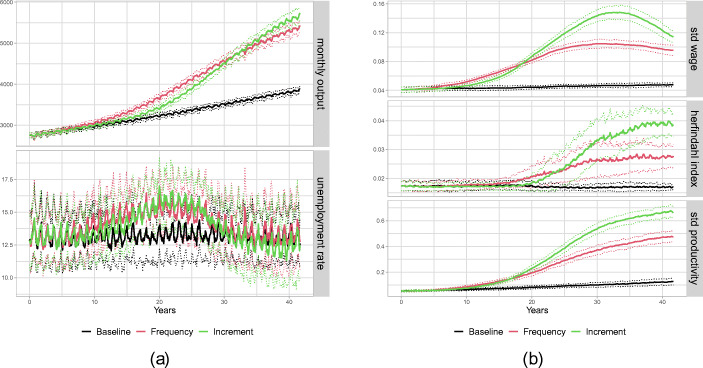
Aggregate dynamics. **(a)** (1) Aggregate output (top) and (2) unemployment (bottom). **(b)** (1) standard deviation for wages (top), (2) Herfindahl index (middle) and (3) standard deviation in firm productivity (bottom)

Although our main agenda is to explore the implications of different types of technological regime shifts on distributional aspects on the household and firm level, we first briefly review how the three considered scenarios compare with respect to aggregate output indicators. In particular, in Fig. 2(a), we depict the time series of aggregate output of the consumption good and the unemployment rate under the three scenarios. As expected, the output grows substantially faster under the new technological regime, compared to the baseline, no matter whether we consider the *Frequency* or the *Increment* scenario. Comparing the two scenarios for the new regime, initially, in particular during the 20-year window of accelerated frontier growth, larger mean output results under the *Frequency* scenario, while in the very long run, the average output under the *Increment* scenario is larger. Throughout the whole considered time span, the distributions of output values under the two scenarios overlap and differences are rather minor. Similar observations apply to unemployment, in the sense that there seems to be a clear difference between the baseline and the scenarios with the new technological regime, but the differences between the *Frequency* and the *Increment* scenario are negligible. In particular, under both scenarios unemployment starts increasing with some delay after the regime change, keeps growing until year 20 when the acceleration of technological change ends, and slowly returns to its baseline value afterwards. These observations indicate that the new technological regime is labour saving in the sense that under the steeper frontier average labour productivity in the economy grows faster than total demand thereby inducing a reduction in employment. Since our focus in this analysis is on the distributional implications of technological change, we do not explore the mechanisms underlying these observations in detail but now turn to the consideration of income and firm size distributions under the different scenarios.

### Aggregate dynamics: increasing polarization

In Fig. 2(b) in the upper panel, we show the inequality dynamics, which we measure by taking the standard deviation across wages, under the three scenarios. It is evident that the acceleration in technological change leads to a strong increase in inequality. However, the patterns of the change in wage distribution differs between the two scenarios. In the *Frequency* scenario, wage inequality reaches its maximum after about 30 years and stagnates thereafter. In contrast, under the *Increment* scenario, inequality increases up to approximately year 35, when it reaches a peak almost twice as large than that under the *Frequency* scenario, but then reverts and begins to decrease such that the gap between the two scenarios narrows considerably until year 40.^5^ As we will discuss below, these patterns are strongly connected to the evolution of firm heterogeneity and the associated industry concentration. Hence, in the middle panel of Fig. 2(b), we show the Herfindahl index,^6^ a common measure for market concentration. It is clearly visible that the shift in the technological regime leads to a stronger concentration of market shares in both scenarios. Similar to the time series of wage inequality, also concentration reaches a higher peak under the *Increment* scenario than under the *Frequency* scenario. However, contrary to wage inequality, market concentration under both scenarios keeps increasing over time although the slope becomes very small as the time interval with accelerated productivity growth moves more and more in the past.

The lowest panel of Fig. 2(b), which shows firm heterogeneity in productivity measured as the standard deviation across firms’ actual productivity, indicates that the increasing concentration is driven by an increasing spread in firm productivity. The patterns for the scenarios are qualitatively very similar to the dynamics of market concentration. Quite intuitively, the increase in the heterogeneity of firm productivity, should lead to increasing heterogeneity of unit costs across firms and therefore induce larger market concentration.

We can summarize our first observations on the aggregate level as follows.

#### **Observation 1**

*The shift in the technological regime leads to increased wage inequality among workers and a stronger dispersion in productivity levels and market shares across firms for both scenarios. In the long run, these effects are more pronounced under the Increment scenario*.

These distributional effects of the regime change keep growing for an extended time interval after the technological growth rate has returned to its baseline level exhibiting strong degrees of hysteresis. Furthermore, the two scenarios show different patterns. First, the effects are stronger for the *Increment* scenario. And second, even though on the firm side both scenarios show an increase in dispersion without any reverting tendency, on the workers side instead wage inequality is decreasing towards the end in the *Increment* scenario. This is absent for the *Frequency* scenario. What are the underlying mechanisms that lead to the observed pattern of polarization in wages as well as in firm productivity and performances? In the next part, we analyse in more depth the distributional aspects of the firm population, which are driving our observations.

### Firm-level dynamics: Two clubs of firms

**Fig. 3:**
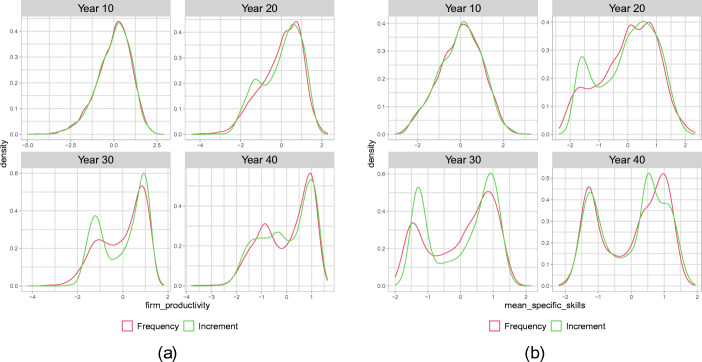
Standardized distribution of firm productivity **(a)** and specific skills **(b)**, pooled over 40 Monte Carlo Simulations, for years 10, 20, 30, 40 after the shift in the technological regime

In Fig. 3(a) we plot the standardized distribution of firm productivities for selected years, pooled across all batch runs in both scenarios. To make the distributions comparable across scenarios and runs, we transform the productivity of firm *i* in run *r* by taking $\frac {A_{i,t,r} - \bar {A}_{t,r}}{\sigma _{t,r}}$ with $\bar {A}_{t,r}$ the mean and *σ*_*t*,*r*_ the standard deviation of productivities in the firm population in run *r* at time *t*. Then, we pool together these individual firm observations over all 40 batch runs. The standardization is done to eliminate systematic differences between runs and distil the evolution of the average shape of productivity distribution in the firm population across runs.^7^

While after 10 years, both scenarios still look quite similar to a Gaussian distribution, after 20 years, the *Increment* scenario develops a second peak. After 30 years this becomes more evident and the distribution of firm productivities displays a bimodal structure with two hubs of firms: leaders and laggards. Interestingly, the distribution looses this shape towards the end in year 40 and the laggard firms become more dispersed. Also, for the *Frequency* scenario, a bimodal structure evolves; however, this change in the shape of the distribution is much slower and becomes bimodal only after 40 years. Hence, the episode of accelerated technological change in years 0–20 in this scenario induces changes in the shape of the firm distribution long after the speed of technological change has returned to its benchmark level. As can be seen from panel (b) of Fig. 3, the emergence of the bimodal productivity distribution is clearly associated by emerging differences across firms in the level of average specific skills of the firms’ employees. The evolution of a bimodal shape of the distribution of specific skills in the firm population precedes the similar dynamics of the productivity distribution and, differently to the productivity distribution, the bimodal shape of the specific skill distribution is persistent until the end of the run also in the *Increment* scenario. We summarize:

#### **Observation 2**

*The shift in the technological regime generates a bimodal structure in the distribution of firm-level productivities and specific skills. Two clubs of firms emerge with leading, high-skilled and high productive firms operating at the technological frontier. In particular with respect to the workers’ specific skills, this effect does not vanish after the regime returns and hence displays a hysteresis effect*

Intuitively, we can explain the emergence of persistent differences between firms with respect to skills and productivity through the interplay of three economic mechanisms captured by the model. First, due to the complementarity between the specific skills of workers and the quality of the firms’ capital goods, for firms with a highly skilled workforce, the return from high-quality vintages is larger and therefore they have higher incentives to invest in (expensive) vintages close to the technological frontier. Second, firms with higher productivity ceteris paribus make higher wage offers and therefore are able to attract workers with high general skills. Third, due to on-the-job learning, the workforce of firms which have high-quality vintages in their capital stock increase their specific skill level faster and this effect is reinforced if workers in these firms on average have higher general skills than the workforce of the less productive competitors. The effect of the interplay of these mechanisms becomes particularly relevant during the time window of accelerated growth of the technological frontier and might lead to an amplification and perpetuation of existing minor, essentially randomly arising, differences between firms at the time of the occurrence of the new technological regime. As will become more clear in our analysis below, firms with heterogeneous productivities continue to co-exist in the long run, because low productivity firms, in spite of their cost disadvantage, based on which they charge higher prices than their competitors, are able to sell positive amounts of the consumption good. Clearly, these are implications of the frictions on labour and consumption good markets captured in our model. Also, as we can see in Figs. 2 and 3, the described processes play out differently in the *Frequency* and the *Increment* scenario. In particular, under the *Increment* scenario, the bimodal shape of the distribution of productivity becomes less pronounced towards the end of the considered time horizon. In the following section, we examine the reasons for these differences in more detail.

### Frequency vs Increment: short and long-run (skill-specific) labour demand

**Fig. 4:**
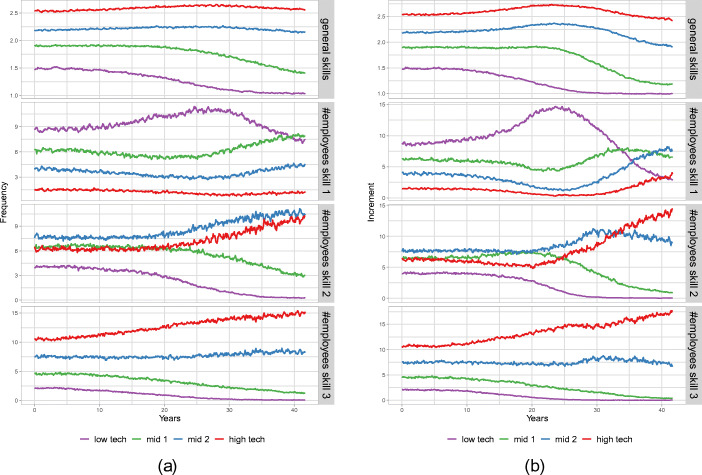
Low-, middle- and high-tech firm groups for **(a)** *Frequency* scenario and **(b)** *Increment* scenario. Panels starting from the top show (1) the average general skill, (2) the number of low-skilled, (3) middle-skilled and (4) high-skilled workers for each tech-group

A main difference between the *Frequency* and *Increment* scenarios is that in the *Frequency* scenario, the monopolistic capital good producer offers a large set of vintages allowing for a more continuous distribution of firm productivities, whereas in the *Increment* scenario only a few options of vintages are available and hence the minimal productivity gap between firms investing on and off the frontier is larger. In light of the self-reinforcing process discussed above, this leads to a faster differentiation of the firm population. To understand this transformation process better, we split the firm population in four different productivity groups. At each point in time, we rank the firms according to their actual productivity and then split the firm population in four equally sized bins. We obtain four tech groups which we call low-, middle 1–, middle 2– and high-tech firms. The 25*%* of firms leading in productivity are represented by the high-tech firm group, whereas firms from the end of the distribution are in the low-tech one. In Fig. 4, we show the four firm groups for the *Frequency* scenario (a) and the *Increment* scenario (b) and plot their average general skills as well as the absolute number of low-, middle- and high-skilled workers in the different panels.

Considering the evolution of the average general skill level of the different types of firms, shown in the upper panels of Fig. 4(a) and (b), we first observe that, consistent with our discussion above, general skills are indeed stratified with respect to firm productivity, i.e. the average general skills are higher in the workforce of more productive firms. Furthermore, in both scenarios the general skill difference becomes more pronounced over time during years 0–25, which includes the entire time window of the accelerated technological change (years 0–20). In particular, during this time, the general skills of the high-tech and mid-tech 2 groups increase whereas those of the mid-tech 1 and low-tech groups decrease. Studying how the number of high-, middle- and low-skill employees evolve over time for the different types of firms, which is shown in the lower three panels of Fig. 4, shows that in both scenarios the less productive firms over time loose the ability to attract workers with high general skills, and, more importantly, the workers with middle general skills, which in the baseline are an important part of the workforce of low productivity firms after year 20, are more and more hired by the high-tech firms.

Overall, in both scenarios we observe that the technological regime change induces a polarization of workers and firms, but restricting attention to the dynamics of general skills qualitative differences between the *Frequency* and the *Increment* scenario arise only after year 25. In the *Increment* scenario, high-tech (and mid tech 2) firms start to substantially increase the number of workers with middle and low skills in their workforce, thereby also reducing their level of average general skills. At the same time the number of high-skilled and middle-skilled workers hired by mid tech 1 firms (i.e. those in the 25–50% quantile region of the productivity distribution) goes to zero in the *Increment* scenario, whereas in the *Frequency* scenario such firms are still able to attract a substantial number of workers outside the lowest skill group.

A first conclusion from this analysis is that the observed qualitative differences between the two scenarios with respect to the shape of the specific skill distribution up to year 20 are not driven by substantial differences in the dynamics of general skill distributions, but rather by the different investment patterns under the two scenarios. As discussed above, in the *Increment* scenario the technological gap between the technological leaders and the firms investing below the frontier widens faster than under the *Frequency* since fewer vintages close to the frontier are available. The earlier polarization of firms with respect to the specific skills in the *Increment* scenario (see Fig. 3(b)) results from the weaker possibilities for on-the-job learning for employees of technological laggards. This is in accordance with the observation that up to year 20 the evolution of wage inequality is quite comparable between the two scenarios. The main qualitative differences in wage inequality between the two scenarios arises after year 20 and hence seems to be associated with the then emerging different patterns of general skill allocations across firms between the two scenarios. Indeed, the crucial difference emerging between the two scenarios is the much stronger increase in market concentration under the *Increment* scenario. The market share of the most productive firms becomes so large that these firms can no longer fulfil their labour demand with workers with high general skills and hence start hiring large numbers of workers with middle general skills and also an increasing number of low-skilled workers.^8^ This implies that an increasing number of low- and middle-skilled workers can profit from the high wages paid by these high productivity firms and since these workers, due to their relatively low general skills, are in the lower part of the wage distribution, this reduces wage inequality. Although the fact that the high-tech firms have to rely partly on workers with low general skills, who are slower on-the-job learners than workers with high general skills, somehow slows down their productivity growth, it does not jeopardize their competitive advantage within the firm population. Since the employees of these firms work with better capital vintages than the employees of technological laggard firms, they still acquire on average higher specific skills than the workers in the low-tech firms, as can be seen from the persistent bimodal distribution of average specific skill levels in firms in the 40 years panel of Fig. 3(b).^9^ Hence, the high market concentration under the *Increment* scenario remains persistently high, explaining the long-run hysteresis effect, while wage inequality decreases during approximately the last 10 years of the run.

Under the *Frequency* scenario, the increase in market concentration is much less pronounced compared to the *Increment* scenario and therefore high-tech firms rely almost completely on high- and middle-skill workers throughout the entire considered time window. Hence, in this scenario, we do not observe any significant decrease of the wage inequality in the last part of the runs. Nevertheless, wage inequality is smaller than in the *Frequency* scenario throughout the entire runs, because, as discussed above, there is less heterogeneity of productivity and the wages a firm pays are proportional to the (general skill specific) productivity of its workers (see Eq. 8).

**Fig. 5:**
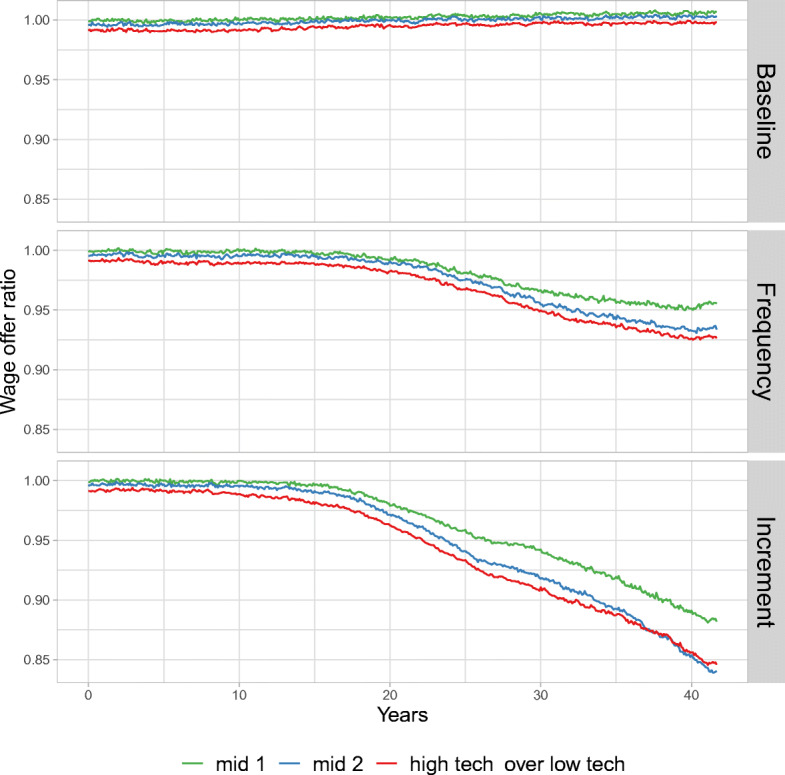
Ratio of base wage offer for high- and middle-tech groups over the low-tech group. Panels show starting from the top the *Baseline*, *Frequency* and *Increment* scenario

To understand why market concentration does not become as high in the *Frequency* scenario as in the *Increment* scenario, it should be noted that although firms endogenously determine their mark-ups the crucial factor determining a firms competitiveness on the market are the unit costs of production, which to a large extend are determined by the wage bill per unit of output. The larger is the difference in the labour costs per unit of output between firms the larger is the market share of the more competitive firms. In light of Eq. 8, it becomes clear that the unit labour costs of a firm essentially depend on its base wage offer $w_{i,t}^{base}$. To study how the relative competitiveness of different types of firms evolve over time in the baseline and the two scenarios of the new technological regime, we show in Fig. 5 the ratio of base wage offers for middle- and high-tech firms divided by the low-tech firm group for all three scenarios. In the *Baseline* scenario, we see homogeneous wage offers and the values fluctuate around 1.0. In contrast, for the *Frequency* scenario all ratios are decreasing over time, indicating that low-tech firms have to increase their base wages to be able to make wage offers that are comparable with their more productive competitors and to attract workers. This effect is however much more pronounced in the *Increment* scenario, under which the firms productivities disperse more strongly. As the high-tech and mid-tech 2 firms gain in market shares and start to target also low-skilled workers, the low-tech firms have to increase the base wage offer considerably in order to be able to still hire low-skilled workers. These increasing base wage offers result however in increasing relative prices of the goods offered by low-tech firms. Hence, additional market shares shift to the more productive firms and the concentration process is reinforced. Nevertheless, due to the frictions on the consumption good market captured in the model, low-tech firms in general can keep positive market shares. Summarizing our analysis, we should distinguish between short and long term effects of the technological regime change.

#### **Observation 3a**

*Although the acceleration of technological change leads in both scenarios also in the short run leads to a larger productivity dispersion across firms and larger wage inequality, general skill allocation across firms and market concentration is hardly affected in the short run*.

All these effects are qualitatively the same under the *Frequency* and the *Increment* scenarios. The key differences between the effects of technological regime change under the two scenarios emerge only in the long run.

#### **Observation 3b**

*In the long run, the two technological regimes diverge due to different underlying dynamics. The Increment* *scenario results in stronger market concentration and higher long run wage inequality compared to the Frequency* *scenario. Also, the dynamics of wage inequality has a non-monotone shape in the Increment* *scenario. Large high-productivity and high-wage market leaders eventually have to rely partly on workers with lower general skills to fulfil their labor demand, which leads to a decreasing wage inequality. Under the Frequency scenario, no such mechanisms arises and concentration and wage inequality stagnate in the long run.*

Under the *Increment* scenario technological laggards face growing unit labour costs needed to be able to fill their vacancies. This reduces their competitiveness, reinforcing market concentration up to a point where all skill groups in the population profit from the high productivity of the high-tech firms. Hence, the economy approaches a state with very high concentration, but decreasing wage inequality. Under the *Frequency* scenario such a self-reinforcing concentration dynamic does not arise.

### The role of heterogeneity of specific skills

**Fig. 6:**
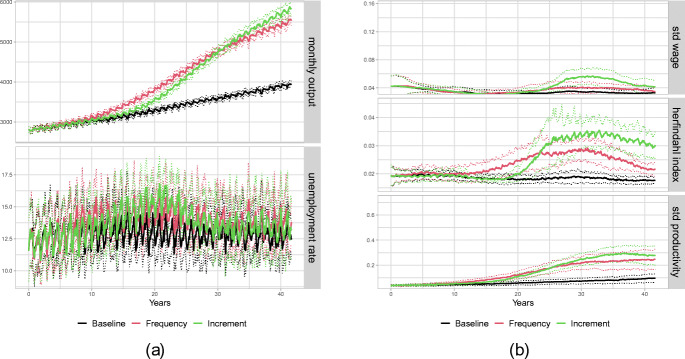
Aggregate dynamics under homogeneous general skills. **(a)** (1) Aggregate output (top) and (2) unemployment (bottom). **(b)** (1) standard deviation for wages (top), (2) Herfindahl index (middle) and (3) standard deviation in firm productivity (bottom)

**Fig. 7:**
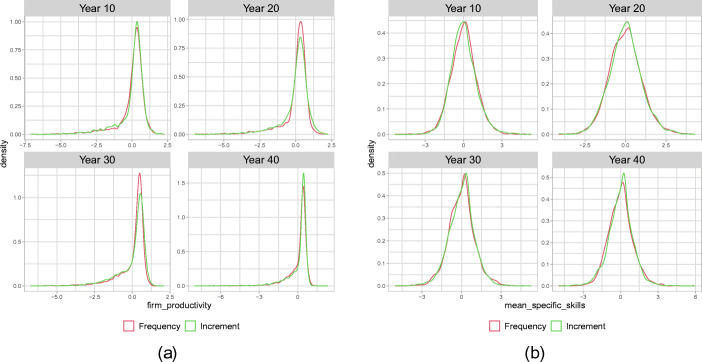
Standardized distribution of firm productivity (**a**) and specific skills (**b**), pooled over 40 Monte Carlo simulations with homogeneous general skills, for years 10, 20, 30, 40 after the shift in the technological regime

An important role in the described mechanisms generating heterogeneity of firm productivities and increasing market concentration is played by the heterogeneity of workers’ general skills and in particular the fact that the observable general skill level can be used by a potential employer as a signal for higher specific skills of a worker. This allows high productivity firms, which pay higher wages, to select employees which are fast learners and on average have above average specific skills, which fosters the emergence of clearly separated technological leaders and laggards. In the absence of such observable heterogeneity between workers the distributional effects of a technological regime change as such and the differences between the *Frequency* and *Increment* scenarios are much smaller. In particular, the long run effects driven by increasing concentration disappear. We illustrate this by showing in Figs. 6 and 7 the equivalent graphs of aggregate dynamics^10^ and evolution of firm distributions to Figs. 2 and 3 with the only exception that we now consider a worker population with homogeneous general skills. More precisely, we consider a scenario in which all workers have general skill level 2. Comparing panels (a) of Figs. 2 and 6 shows that as far as economic growth and unemployment dynamics go this change in the skill distribution has only negligible effects. Quite on the contrary, the comparison of panel (b) highlights that the distributional effects under homogeneous general skills do not only differ quantitatively but also qualitatively from those in our default scenario. Considering first the short run effects, it can be observed that in year 20 the levels of market concentration and standard deviation of firm productivity are comparable to that in the baseline scenario, whereas the level of wage inequality is much smaller and actually does not seem to change significantly relative to the level prior to the technological regime change. Concerning the long run effects, we observe that after year 20, similarly to the benchmark case with heterogeneous general skills, concentration increases sharply under the *Increment* scenario. However, under homogeneous general skills this initial increase, driven by the increased gap between available vintages, is not reinforced through the sorting of most productive workers to most productive firms and stops soon after the speed of the technological change has returned to its benchmark level. As can be seen in Fig. 7, no bimodal firm distribution with technological leaders and laggards arises and, although also in this case we observe hysteresis in the sense that the heterogeneity of firm productivity stays at a level that is larger than in the baseline without the technological regime change, the long run effect on firm heterogeneity is much smaller than under heterogeneous general skills and also the difference between the *Frequency* and *Increment* scenarios is much smaller. We can formulate a final observation.

#### **Observation 4**

If workers are homogenous with respect to their general skills, the observed polarization patterns are strongly mitigated. No bimodal firm distribution with technological leaders and laggards arises. This highlights the pivotal role played by the heterogeneity of workers’ skills.

## Conclusion

In this paper, we study distributional effects of a technological regime change, which, in accordance with standard insights about technological trajectories, induces an acceleration of the speed of change of the technological frontier for a limited time window. Using a framework incorporating heterogeneous workers and firms as well as endogenous technology choices of firms and on-the-job learning of workers, we examine how these effects emerge over time and in how far they differ between scenarios in which the new technological regime is characterized by more frequent respectively more substantial productivity increasing innovations compared to the baseline regime. Our approach allows to capture the co-evolution of the industry structure, the firms’ technological choices, the workers skill distribution and (firm specific) demand in a closed agent-based macroeconomic model.

A key insight from our analysis is that in particular the long run effects of the regime change depend crucially on the type of the technological change process. If the frontier moves along the technological trajectory in a few large steps, giving rise to a sparse set of technological choices for production firms using the technology (the *Increment* scenario), an increasing and persistent polarization of firms emerges with a strong sorting of most productive workers to technologically leading firms. Market concentration keeps increasing in this scenario with the high-tech firms gaining larger and larger market shares, but the effect of this process on wage inequality is ambivalent. Whereas initially the increasing heterogeneity of firm productivity translates into increasing wage inequality, in the long run, wage inequality decreases because workers in the lower part of the wage distribution start profiting from the increasing productivity of high-tech firms. The reason for this effect is that due to their large market shares the firms in the upper part of the productivity distribution cannot fulfil their labour demand with high-skilled workers. Hence, more low-skilled workers become employed by high-tech firms and also the increased competition on the labour market pushes up wages. If technological change occurs in many small innovation steps along the technological trajectory (the *Frequency* scenario) the induced market concentration as well as the emerging firm heterogeneity is substantially smaller compared to the *Increment* scenario; however, the gap in the resulting wage inequality between the two scenarios decreases over time and in the very long run inequality is larger in the scenario where technological change happens in many small steps. A second key insight from our analysis is that, in order to understand the distributional implications of different types of technological change processes, it is crucial to capture explicitly the heterogeneity of worker characteristics and also the observability of these characteristics for potential employers.

These insights do not only shed light on the important relationship between the nature of processes of technological change and the resulting effects on inequality, but also point to a potentially ambiguous role of market concentration for the evolution of wage distribution. In particular, they highlight that in settings characterized by complementarity between capital quality and worker skills, but potential substitutability between different skill groups, concentration of large market shares at a small group of highly productive firms might reduce wage inequality. This insight raises interesting questions on the role of industrial and competition policy from a distributional perspective. Exploring these issues in more detail is a promising avenue for future work.

## Appendix A: Technical details

The parameter values are based on Dawid et al. (2019) and are summarized in Table 2. The number of agents and the distribution of skills across workers are displayed in Table 3. The initialization of variables is the same as in Dawid et al. (2019).

**Table 2:** Values of selected parameters

Parameter	Description	Value
*λ*	Bargaining power of the capital goods producer	0.5
*δ*	Capital depreciation rate	0.01
*γ*^*v*^	Logit parameter for vintage choice	30.0
*u*	Wage replacement rate	0.70
*φ*	Firm base wage update	0.01
*ψ*	Reservation wage update	0.01
*ν*	Number of unfilled vacancies triggering wage update	2
*α*_*D*_	Number of applications per day	3
*α*_*T*_	Total number of applications per month	5
*γ*^*c*^	Intensity of consumer choice	17
*χ*	Service level for the expected demand	0.8
*ρ*	Discount rate	0.02
*S*	Firm time horizon in months	24
Φ	Target wealth/income ratio	16.67
*κ*	Adjustment wealth/income ratio	0.01
*r*^*c*^	ECB interest rate	0.05

**Table 3:** Distribution of agents and skills

Agents		Value	
Households		1600	
Consumption good firms		80	
Capital good producer		1	
Banks		20	
Skill distribution	Low	Middle	High
General skill level *b*_*g**e**n*_	1	2	3
Percentage of households	33.3*%*	33.3*%*	33.3*%*
Adaption speed specific skills $\chi \left (b^{gen}_{h}\right )$	0.0125	0.024765	0.03703

Our results are based on 40 Monte Carlo runs for each scenario. To avoid differences across scenarios stemming from different initial conditions in year 0, in which the trajectories diverge, we create 40 snapshots with different random seeds. Then, we use these to start each scenario at year 0 with the same starting points.

## Appendix B: Statistical tests

We provide the results of statistical tests across the three scenarios confirming statements made in Section 4. We use a Mann-Whitney *U*-test, the non-parametric counterpart of the *t*-test for unpaired samples, with equality of the two scenarios as the Null-hypothesis. In Table 4, we document ratios between scenarios and in brackets below *p*-values (all p-values below 5% are stated in bold).

**Table 4:** Mann-Whitney *U*-test across scenarios (corresponding to Fig. 2(**a**,**b**))

		Ratios (*p*-values) for year			
	Variables	10	20	30	40
Frequency over baseline					
	Monthly output	1.03	1.13	1.29	1.40
		(**0.000**)	(**0.000**)	(**0.000**)	(**0.000**)
	Unemployment rate	1.06	1.17	1.08	0.94
		(**0.049**)	(**0.000**)	(**0.015**)	(0.054)
	Standard deviation wage	1.21	1.85	2.25	2.05
		(**0.000**)	(**0.000**)	(**0.000**)	(**0.000**)
	Herfindahl index	1.02	1.18	1.58	1.63
		(0.450)	(**0.000**)	(**0.000**)	(**0.000**)
	Standard deviation productivity	1.31	2.38	3.67	3.78
		(**0.000**)	(**0.000**)	(**0.000**)	(**0.000**)
Increment over baseline					
	Monthly output	1.01	1.05	1.28	1.45
		(0.135)	(**0.000**)	(**0.000**)	(**0.000**)
	Unemployment rate	1.01	1.22	1.02	0.94
		(0.683)	(**0.000**)	(0.160)	(0.201)
	Standard deviation wage	1.08	1.97	3.09	2.59
		(**0.000**)	(**0.000**)	(**0.000**)	(**0.000**)
	Herfindahl index	1.00	1.05	1.97	2.29
		(0.958)	(0.141)	(**0.000**)	(**0.000**)
	Standard deviation productivity	1.16	2.78	5.25	5.30
		(**0.000**)	(**0.000**)	(**0.000**)	(**0.000**)
Increment over Frequency					
	Monthly output	0.98	0.93	0.99	1.04
		(**0.000**)	(**0.000**)	(0.133)	(**0.000**)
	Unemployment rate	0.95	1.04	0.95	1.02
		(0.136)	(0.258)	(0.206)	(0.683)
	Standard deviation wage	0.90	1.07	1.37	1.26
		(**0.000**)	(**0.000**)	(**0.000**)	(**0.000**)
	Herfindahl index	0.98	0.89	1.25	1.41
		(0.368)	(**0.002**)	(**0.000**)	(**0.000**)
	Standard deviation productivity	0.89	1.17	1.43	1.40
		(**0.000**)	(**0.000**)	(**0.000**)	(**0.000**)

## Footnotes

1 This expansion in firm heterogeneity has been termed *neodualism* (Dosi et al. 2019) or the *great divergence* (Berlingieri et al. 2017).

2 Note that we consider a version of the model with a single integrated economic region.

3 Note that this implies that in the following experiments the end of the burn-in phase is labelled as period *t* = 0.

4 Note that specific skills *b*_*h*,*t*_, like the average quality of the capital stock *A*_*i*,*t*_, are measured in terms of productivity units.

5 If we consider the dynamics beyond year 40, we observe that starting approximately in year 47, the wage inequality under the *Increment* scenario is actually lower than that under the *Frequency* scenario. The corresponding simulation results are available from the authors upon request.

6 The HHI is calculated as the sum of squared market shares ${\sum }_{i} {s_{i}^{2}}$. Note that in our case with 80 firms, equally distributed market shares would result in a HHI of 0.0125.

7 It should be noted that due to this procedure the fact that the spread of the distributions depicted in Fig. 3 is similar between the two scenarios does not contradict our observation from Fig. 2(b) that the standard deviation of firm productivity is substantially larger under the *Increment* than under the *Frequency* scenario. More general, only the shape of the distribution can be interpreted in a meaningful way, whereas the range of the distribution is determined by the standardization and should not be compared across the different panels.

8 Note here that firms expand to maximize their profits, but since firms cannot simply hire a worker with the desired high general skill level and need to go through the decentralized matching process on the labour market, this expansion might come at the cost of reducing the average general skill level within the workforce.

9 Clearly, the substitutability of workers across general skills drives this result. If high-tech firms were required to hire only high-skilled labour, they would be constrained by the skill shortage. Having skill substitutability in the model can be interpret as internal training programs, which high productive firms use to counteract a shortage of skilled labour.

10 Note that we choose the same limits for the *y*-axis to visualize the differences.
